# Supplementary material for: Phosphate Adsorption from Aqueous Solution Using Electrospun Cellulose Acetate Nanofiber Membrane Modified with Graphene Oxide/Sodium Dodecyl Sulphate
Source: Membranes (Basel). 2021 Jul 20;11(7):546. doi: 10.3390/membranes11070546 (PMC8307572; doi:10.3390/membranes11070546)
Supplement: Supplementary file 1 [file membranes-11-00546-s001.zip › membranes-1283517-supplementary.pdf]

$$C_e/q_e = 1/q_m b + C_e/q_m \quad \text{(S1)}$$

where, q_m and b are the Langmuir adsorption constant related to maximum monolayer adsorption capacity and energy, respectively.

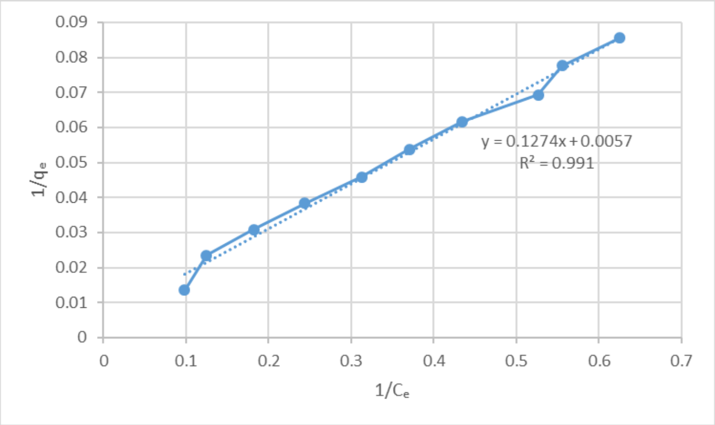

Figure S1. A Langmuir plot ($1/q_e$ vs. $1/C_e$) for phosphate adsorption by CA/GO/SDS at different masses of membrane.

Table S1. Constant parameters and correlation co-effecients calculated for different adsorption isotherm models for phosphate adsoption.

**Isotherm**	**Mass of membrane (g)**	**constants**			
Langmuir		Q_o	*b*	R_L	R^2
	0.05	175.44	0.04	0.5	0.991
Freundlich		K_f	*n*		
	0.05	7.647	1.117		0.9774
